# Resveratrol ameliorates mitochondrial biogenesis and reproductive outcomes in women with polycystic ovary syndrome undergoing assisted reproduction: a randomized, triple-blind, placebo-controlled clinical trial

**DOI:** 10.1186/s13048-024-01470-9

**Published:** 2024-07-10

**Authors:** Negar Ajabi Ardehjani, Marzieh Agha-Hosseini, Maryam Shabani Nashtaei, Mahshad Khodarahmian, Maryam Shabani, Masoome Jabarpour, Farzane Fereidouni, Tayebeh Rastegar, Fardin Amidi

**Affiliations:** 1 Department of Anatomy, School of Medicine, Tehran University of Medical Sciences, Tehran, Iran; 2 Department of Infertility, Shariati Hospital, Tehran University of Medical Sciences, Tehran, Iran; 3 Department of Infertility, Arash Women’s Hospital, Tehran University of Medical Sciences, Tehran, Iran; 4 Department of Clinical Biochemistry, School of Medicine, Tehran University of Medical Sciences, Tehran, Iran; 5 Department of Infertility, Yas Hospital, Tehran University of Medical Sciences, Tehran, Iran

**Keywords:** Resveratrol, Polycystic ovary syndrome, Mitochondrial biogenesis, Granulosa cells, Antioxidants, Assisted reproductive technique

## Abstract

**Background:**

This study was designed to examine the effect of resveratrol on mitochondrial biogenesis, oxidative stress (OS), and assisted reproductive technology (ART) outcomes in individuals with polycystic ovary syndrome (PCOS).

**Methods:**

Fifty-six patients with PCOS were randomly assigned to receive 800 mg/day of resveratrol or placebo for 60 days. The primary outcome was OS in follicular fluid (FF). The secondary outcome involved assessing gene and protein expression related to mitochondrial biogenesis, mitochondrial DNA (mtDNA) copy number, and adenosine triphosphate (ATP) content in granulosa cells (GCs). ART outcomes were evaluated at the end of the trial.

**Results:**

Resveratrol significantly reduced the total oxidant status (TOS) and oxidative stress index (OSI) in FF (*P* = 0.0142 and *P* = 0.0039, respectively) while increasing the total antioxidant capacity (TAC) (*P* < 0.0009). Resveratrol consumption also led to significant increases in the expression of critical genes involved in mitochondrial biogenesis, including peroxisome proliferator-activated receptor gamma coactivator (PGC-1α) and mitochondrial transcription factor A (TFAM) (*P* = 0.0032 and *P* = 0.0003, respectively). However, the effect on nuclear respiratory factor 1 (Nrf-1) expression was not statistically significant (*P* = 0.0611). Resveratrol significantly affected sirtuin1 (SIRT1) and PGC-1α protein levels (*P* < 0.0001 and *P* = 0.0036, respectively). Resveratrol treatment improved the mtDNA copy number (*P* < 0.0001) and ATP content in GCs (*P* = 0.0014). Clinically, the resveratrol group exhibited higher rates of oocyte maturity (*P* = 0.0012) and high-quality embryos (*P* = 0.0013) than did the placebo group. There were no significant differences between the groups in terms of chemical or clinical pregnancy rates (*P* > 0.05).

**Conclusions:**

These findings indicate that resveratrol may be a promising therapeutic agent for patients with PCOS undergoing assisted reproduction.

**Trial registration number:**

http://www.irct.ir; IRCT20221106056417N1; 2023 February 09.

## Introduction

PCOS is a multifactorial endocrine disorder that affects approximately 8–15% of reproductive-aged women [1]. Recent research has provided new insights into irregular mitochondrial indices in patients with PCOS [2]. The abundance of mitochondria in the granulosa cells (GCs) surrounding a specific oocyte is positively correlated with oocyte maturation, fertilization, and successful embryo development [3]. The mitochondrial biogenesis pathway is a fundamental factor in follicular development [4]. Mitochondrial biogenesis involves the expansion and division of existing mitochondria [5]. It seems that oxidative stress (OS) is caused by insufficient physiological antioxidants to counterbalance excess reactive oxygen species (ROS), which are present in individuals with PCOS [6]. A previous study demonstrated that changes in mitochondrial biogenesis might contribute to hyperandrogenism, while an increase in mitochondrial biogenesis could improve PCOS by reducing ROS in GCs [7]. These findings highlight that mitochondrial dysfunction plays a critical role in the progression and management of PCOS [8]. The literature frequently mentions that the mtDNA copy number is a marker of mitochondrial biogenesis and mitochondrial content [1]. A study demonstrated that impaired mitochondrial function, characterized by decreased mtDNA copy numbers and mutations in mitochondrial genes, might contribute to the progression of PCOS [9]. The transcriptional peroxisome proliferator-activated receptor gamma coactivator (PGC-1α) collaborates with nuclear respiratory factor 1 (Nrf-1), promoting the expression of mitochondrial transcription factor A (TFAM), which directly regulates the replication and transcription of mtDNA. The activation of the TFAM promoter plays a vital role in the transcription of nuclear and mitochondrial genes, serving as a mechanism for mitochondrial biogenesis [10]. Sirtuin 1 (SIRT1) is an NAD+-dependent deacetylase that interacts directly with PGC-1α, resulting in the upregulation of PGC-1α expression [11]. The specific mechanisms linking abnormal mitochondrial markers to the development of PCOS remain unclear. Consistent with this notion, it is imperative to research the molecular processes of PCOS to identify potential therapeutic targets. Recently, there has been a significant focus on antioxidant treatment as a natural and low-risk supplement in medical research for mitigating the impact of PCOS on female fertility [12]. Resveratrol is a natural polyphenolic compound identified as 3-5-4′-trihydroxystilbene that is found in various plant-derived sources, such as grapes, berries, and peanuts [13]. Resveratrol plays a critical role in the regulation of antioxidant enzymes and the inhibition of prooxidant factors [14]. Resveratrol has been used as an activator of SIRT1 to regulate mitochondrial activity in both animals and cell lines [15, 16]. Additionally, numerous clinical trials have suggested that resveratrol supplementation may alleviate symptoms of PCOS [17–19]. Furthermore, previous research has revealed that the impact of resveratrol on the mitochondrial biogenesis of GCs may explain the favorable effects of this polyphenol on the female reproductive system, suggesting potential therapeutic implications in clinical practice [20]. This clinical trial aimed to explore the potential of resveratrol as a therapeutic agent for improving mitochondrial biogenesis and assisted reproductive technique (ART) outcomes in patients with PCOS.

## Materials and methods

### Trial design

This study was a randomized, triple-blind, placebo-controlled trial that evaluated the effects of resveratrol in patients with PCOS. The present study was registered on the Iranian website for registration as a clinical trial (2023 February 09; Registration Code: IRCT20221106056417N1; http://www.irct.ir) before enrolling the first patient. This paper presents some of the outcomes from clinical trials registered with IRCT. Additionally, this study received approval from the Ethics Committee of Tehran University of Medical Sciences, Shariati Hospital, Tehran, Iran (2023 January 07; Ethics Code: IR.TUMS.SHARIATI.REC.1401.021). All participants provided informed consent.

### Participants

#### Inclusion criteria

The Rotterdam criteria define four phenotypes of PCOS: (A) androgen excess, ovulatory dysfunction, and polycystic ovary morphology (PCOM) on ultrasound; (B) androgen excess and ovulatory dysfunction; (C) androgen excess and PCOM; and (D) ovulatory dysfunction and PCOM [21]. The International Evidence-Based Guideline [22] supports these criteria, stating that the diagnosis requires the presence of at least two of the following criteria in adult women: oligo-anovulation, androgen excess (either clinical or biochemical), and ultrasound assessment of ovarian morphology [23]. The inclusion criteria were as follows: (i) 18‒35 years of age; (ii) patients who were diagnosed with PCOS based on the Rotterdam criteria; and (iii) undergoing intracytoplasmic sperm injection (ICSI) treatment at the Department of Omid Fertility Clinic in Tehran, Iran.

#### Exclusion criteria

The exclusion criteria were as follows: (i) patients with hyperprolactinemia, thyroid disease, congenital adrenal hyperplasia, or Cushing’s syndrome (identified based on the patient’s medical history); (ii) individuals with severe endometriosis (stages 3 and 4 according to the revised AFS-rAFS classification) [24], ovarian tumors, or female infertility factors unrelated to cervical and tubal issues; (iii) those with severe obesity (defined as a body mass index (BMI) > 35); (iv) patients with follicle-stimulating hormone (FSH) levels exceeding 10 mg/mL; (v) individuals with systemic disorders such as metabolic syndrome, diabetes, hyperlipidemia, and cardiovascular disease; (vi) patients who became pregnant, or those who were using any medications affecting reproductive physiology within three months prior to the study; (vii) patients who smoke and/or consume alcohol; (viii) individuals with any autoimmune disease; and (ix) those with severe male factor infertility, particularly nonobstructive azoospermia.

### Randomization and blinding

Random allocation was performed through a 1:1 allocation ratio with a balanced block randomization approach, with block sizes of 4 containing letters A and B (representing the resveratrol and placebo groups). The contents were concealed using sequentially numbered sealed opaque envelopes. Patient allocation was conducted by an independent statistician using online platforms. Researchers then enrolled patients, and a gynecologist, who remained blinded throughout the study, diagnosed patients with PCOS based on the Rotterdam criteria before assigning participants to interventions. Researchers, patients, and statisticians were blinded throughout the study and treatment allocation. The appearance of the placebo, the resveratrol capsules, and the packaging were identical to ensure blinding, and the capsules were provided by the same company simultaneously.

### Intervention

The effective dose and duration of resveratrol supplementation were derived from previous studies [18, 19, 25]. Based on their findings, a daily dosage of 800 mg/day (2 × 400 mg capsules) of resveratrol (transresveratrol, 99% purified, Mega Resveratrol, UK) was orally administered to patients in the treatment (resveratrol) group, and patients in the placebo group received placebo capsules (maltodextrin and lactose) every day for 60 days before oocyte collection. The shape, scent, and color of the placebo capsules were identical to those of the resveratrol capsules. The intervention and placebo groups were administered two tablets at the total daily dose. Also, it is crucial to control for confounding factors like, physical activity, and food intake. All eligible patients were instructed to maintain their usual lifestyle, including their level of physical activity throughout the study, and to avoid nutritional supplements or foods high in resveratrol, which were listed for the patients. Additionally, patients had to notify the researchers of any changes in their diet. To more closely monitor the effect of resveratrol supplementation, the International Physical Activity Questionnaire-Short Form (IPAQ SF) was used to estimate the level of physical activity at baseline and at the end of the intervention. The questionnaire recorded self-reported activity at four levels: vigorous physical activity (e.g., heavy lifting), moderate physical activity (e.g., carrying light loads), walking, and sitting [26]. To assess participants’ compliance and adherence to the trial protocol, the researcher made telephone calls every 15 days to evaluate capsule consumption and any new symptoms or side effects since the last interview.

#### Ovarian stimulation procedure

All patients underwent a controlled ovarian stimulation regimen known as the flexible antagonist regime. From the third day of the menstrual cycle, 150–300 IU of rFSH (Gonal-F, Merck Serono SA, Switzerland) was given, with the optimum dosage tailored based on the estradiol concentration and ovarian response. Ovarian monitoring was conducted, and once at least two follicles measuring 14–15 mm in size were observed, the gonadotropin-releasing hormone antagonist cetrorelix acetate cetrotide (0.25 mg/day; Merck Serono SA, Switzerland) was administered. A total of 10,000 IU of human chorionic gonadotropin (hCG) (Ovitrelle, Merck Serono SA, Switzerland) was used as the trigger once at least two follicles reached a diameter of ≥ 18 mm, and cetrotide consumption was discontinued. At 36-hour intervals, each follicle was measured immediately before aspiration, and subsequently, oocyte retrieval was performed under transvaginal ultrasound guidance [27].

### Outcomes

The demographic and clinical data of the patients, such as age, BMI, duration of infertility, menstruation, and menstrual cycle duration, were documented. Additionally, the hormonal profiles of patients during the follicular phase (including serum levels of FSH, luteinizing hormone (LH), prolactin (PRL), thyroid stimulating hormone (TSH), testosterone, and anti-Müllerian hormone (AMH)) were evaluated. The primary outcome was the change in OS markers in FF. At the same time, the levels of genes and proteins, as well as the mtDNA copy number and adenosine triphosphate (ATP) content, in GCs and ART outcomes were recorded and subjected to analysis as secondary outcomes.

#### Sample collection

Following the methods of a previous study [27], individual samples comprising FF and GCs were collected from each patient. The FFs obtained from follicles with a diameter of at least 16 mm were pooled together and immediately subjected to centrifugation at 3000×g for 15 min at room temperature. The resulting supernatant was preserved at − 80 °C until analysis. The sediments were resuspended in 3 mL of phosphate-buffered saline (PBS; Sigma, Germany), gently layered onto 5 mL of Ficoll-Hypaque (Lymphodex, Inno-Train, Germany), and centrifuged at 400×g for 20 min at room temperature to eliminate red blood cells and debris. The GCs were retrieved from the layer at the gradient interface, centrifuged twice at 600×g for 5 min at room temperature with 1 mL of PBS (Sigma, Germany), and stored at − 80 °C until RNA and protein extraction.

#### Follicular fluid analysis

The levels of OS markers (total antioxidant capacity [TAC] and total oxidant status [TOS]) in the FF samples were evaluated twice for accuracy using readily available NaxiferTM and NatosTM Kits provided by Navand Salamat in Tehran, Iran. Furthermore, the oxidative stress index (OSI), determined by dividing the TOS by the TAC, was assessed in both groups and is expressed in µmol of H2O2/mmol of Fe2+.

#### Gene expression analysis by qRT‒PCR

Total RNA was extracted from frozen GCs using a column method (RNA Extraction Kit, AnaCell, Iran) following the manufacturer’s instructions. The concentration and purity of the isolated RNA were assessed using a spectrophotometer (Biochrom). Subsequently, complementary DNA (cDNA) synthesis was performed using a cDNA reverse transcription kit (cDNA Synthesis Kit, AnaCell, Iran). Quantitative real-time polymerase chain reaction (qRT–PCR) analysis was performed using SYBR Green RealQ Plus 2× Master Mix Green (Amplicon) on a Real-Time ABI^®^ Step One thermocycler. The housekeeping gene glyceraldehyde-3-phosphate dehydrogenase (GAPDH) was used for normalization, and the relative expression of each gene was determined using the 2 ^−^ ^ΔΔCt^ method. For real-time PCR analysis, 24 samples from each group were used. The primer sequences utilized in the study are detailed in Table 1.

**Table 1:** Designed sequences of primers used for qRT‒PCR and qPCR

Nuclear Gene	Primer sequences (5’ → 3’)	Accession number	Product length
*PGC-1α*	F: GCTCTCGTTCAAGGTC R: GTGCGGTGTCTGTAG	NM_001330751.2	139
*Nrf-1*	F: ATGGCACTGTCTCACTTATC R: TCTCACCTCCCTGTAACG	NM_005011.5	162
*TFAM*	F: CTAAAGAACAACTACCC R: TATACCTGCCACTCC	NM_003201.3	148
*GAPDH*	F: CGCCAGCCGAGCCACATC R: CGCCCAATACGACCAAATCCG	NM_002046.7	76
Mitochondrial Gene			
*ND1*	F: ACACCTCTGATTACTCCTG R: CCTGCGGCGTATTCG	NC_012920.1	144
*Beta-actin*	F: GGTCCTCACTGCCTGTC R: TCGTCATACTCCTGCTTGC	NC_000007.14	139

qRT–PCR, Quantitative real-time polymerase chain reaction; qPCR, Quantitative polymerase chain reaction; PGC-1α, Peroxisome proliferator-activated receptor gamma coactivator; Nrf-1, Nuclear respiratory factor 1; TFAM, Mitochondrial transcription factor A; ND1, NADH dehydrogenase subunit 1; GAPDH, glyceraldehyde-3‐phosphate dehydrogenase

#### Western blot

Total protein was extracted from frozen GCs using RIPA buffer. The protein concentration was determined using a Bradford protein quantification kit (DNAbiotech, Iran) following the manufacturer’s instructions. Equal amounts of protein samples were then separated by SDS‒PAGE and transferred onto an immune-Blot™ polyvinylidene difluoride (PVDF) membrane (Bio-Rad Laboratories, CA, USA; Cat No: 162–017777). Next, the membranes were blocked with 5% bovine serum albumin (BSA) (Sigma Aldrich, MO, USA; Cat No: A-7888) in 0.1% Tween 20 for 1 h. Subsequently, the blots were incubated with specific antibodies against total SIRT1 (Abcam, Cat No: ab189494) and PGC1-α (Abcam, Cat No: ab191838) for 1 h at room temperature. For normalization, β-actin (Abcam, Cat No: ab8227) was used as the internal control. Finally, the membrane was incubated with a goat anti-rabbit IgG H&L (HRP) (Abcam, Cat No: ab6721) secondary antibody. The membrane was subsequently subjected to enhanced chemiluminescence (ECL) for 1–2 min. The densitometry of each band was determined using the gel analyzer version 2010a software (NIH, USA). This process allowed the analysis and comparison of protein expression levels. For western blot analysis, six samples from each group were used.

#### Measurement of mitochondrial copy number

Total DNA (genomic and mitochondrial) was extracted from GCs using a column method according to the manufacturer’s instructions (DNA Extraction Kit, AnaCell, Iran). mtDNA copy number relative to genomic DNA content was determined based on the ratio of the mtDNA-encoded NADH-dehydrogenase subunit 1 (ND1) gene to β-actin as a nuclear endogenous control region using quantitative polymerase chain reaction (qPCR) assays. mtDNA quantification was performed using the ΔΔCt method, in which all the mtDNA target (ND1) Ct values were normalized to the β-actin values [28, 29]. For the qPCR analysis, 24 samples from each group were used. The primer sequences are outlined in Table 1.

#### Intracellular ATP level measurement

The ATP concentration in the GCs was measured via an ATP colorimetric assay kit (Adenosine Triphosphate ELISA Kit, Abbexa, Cat. No. abx574124). The absorbance of the samples was measured using an ELISA plate reader set to 450 nm. ATP concentrations in the samples were determined by comparison to a standard curve. Twenty-four samples from each group were used for analysis.

#### Clinical follow-up

Following oocyte retrieval, the cumulus cells were eliminated through hyaluronidase enzyme (Sigma^®^, USA) and delicate mechanical pipetting. Then, the oocytes were categorized into three groups based on their nuclear maturity profile immediately after the removal of cumulus cells: (I) MII oocytes, featuring the presence of the first polar body; (II) MI oocytes lacking the first polar body; and (III) germinal vesicle oocytes containing a prominent nucleus within the cytoplasm. The total number of harvested oocytes and the oocyte maturation rate were documented. The oocyte maturation rate was determined using the following formula: rate of maturity oocyte = (number of MII stage oocytes/total oocyte count) ×100% [30].

Injectable MII-stage oocytes were utilized for the ICSI procedure. Sixteen to nineteen hours after the injection, the presence of two pronuclei (2PN) confirmed successful fertilization. The fertilization rate was determined using the following formula: fertilization rate = (2PN/total MII oocyte count) ×100% [31].

Two to three days after ICSI, information on embryo development, including the number of embryos and high-quality embryo rate, was recorded. High-quality embryos were defined as those exhibiting Grade A or B cleavage based on Association for the Study of Reproductive Biology (ASEBIR) standards, which considered factors such as the number of cells (blastomeres), the extent of embryo fragmentation, cell symmetry, and the presence of multinucleation, vacuoles, and pitting relative to low-quality embryos. The high-quality embryo rate was determined as the percentage of high-quality embryos relative to the total number of embryos [32]. Consistent with the standard clinical approach, which aims to mitigate the risk of ovarian hyperstimulation syndrome (OHSS) and enhance overall clinical results, embryos were cryopreserved on days 3 and 5. Subsequently, 2 or 3 embryos were transferred in the following two cycles. Patient follow-up included the computation of chemical and clinical pregnancy rates, which served as the key outcomes of the randomized clinical trial. The chemical pregnancy rate was determined as the ratio of the serum β-hCG level 14 days after embryo transfer to the total number of embryo transfer cycles [33]. Moreover, the clinical pregnancy rate was determined by the presence of a gestational sac and fetal heartbeat observed during an ultrasound scan at approximately 6–7 weeks of gestation divided by the total number of embryo transfer cycles [34].

### Statistics

#### Sample size calculation

The sample size calculation was estimated from a previous study [35], with a significance level of 0.05 and 80% power to detect a 17% increase in the mean FF TAC as the primary outcome in the intervention group compared to the placebo group, while accounting for a potential 10% patient dropout rate during the study, the final sample size was 56 (28 patients in each group).

#### Statistical methods

The statistical analysis was conducted with SPSS 26.0, and GraphPad Prism 9.0 software was used for data generation. Normally distributed data were tested using the Kolmogorov‒Smirnov test. The differences between the resveratrol and placebo groups were assessed using an Unpaired t test. Nonnormally distributed data were analyzed using the Mann–Whitney test. Chemical and clinical pregnancies were evaluated using Fisher’s exact test. A significance level of *P* < 0.05 indicated statistical significance for all the statistical assessments. All experimental data are presented as the means and standard deviations (SDs) or as medians (25th–75th percentiles).

## Results

**Fig. 1:**
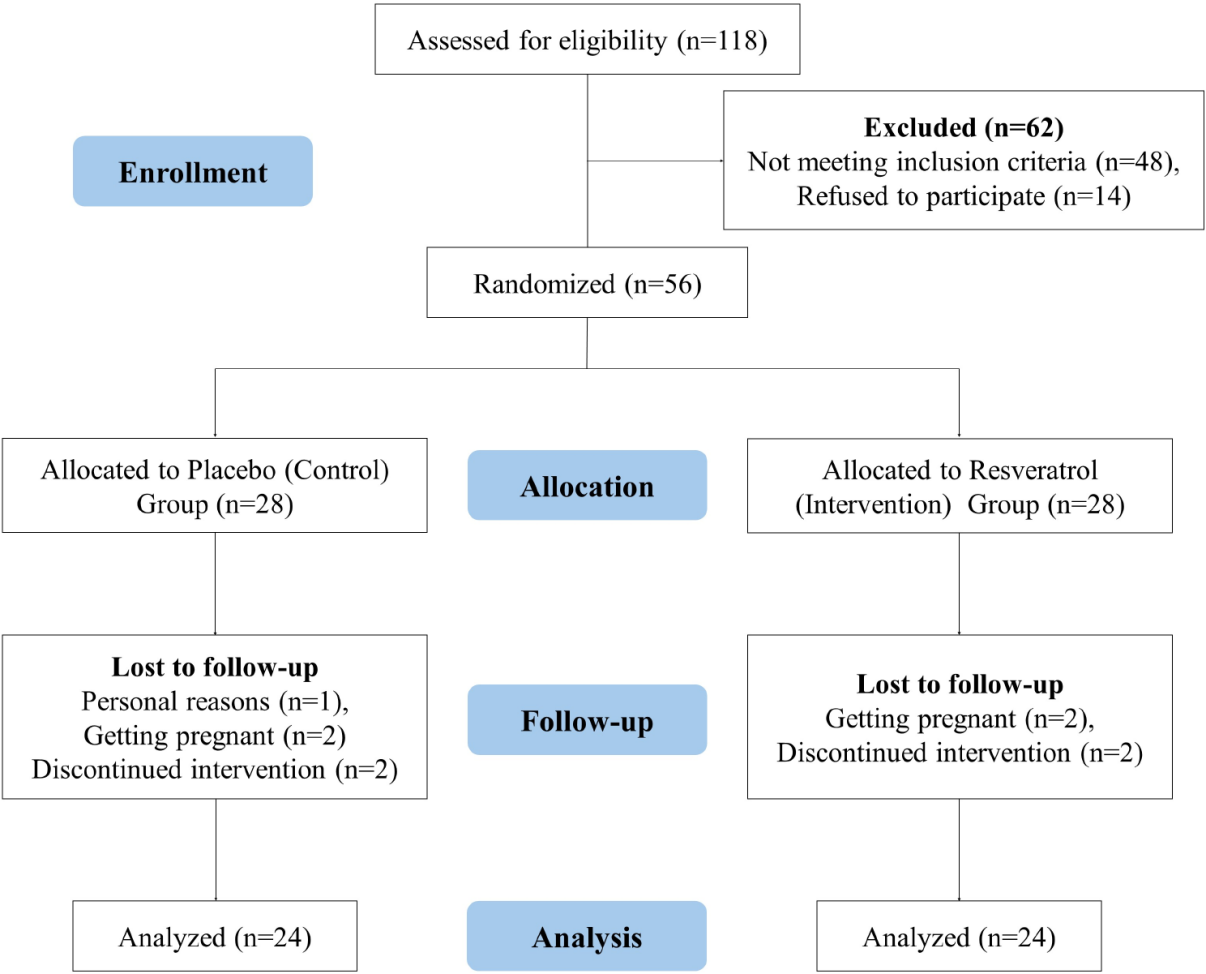
Enrollment, allocation, and follow-up of participants and the CONSORT diagram of the clinical trial

The study was conducted from February 2023 to December 2023. Resveratrol was well tolerated throughout the treatment, with no patients reporting any side effects. Initially, 118 participants were screened, and 62 (52.5%) were subsequently excluded. The remaining 56 (47.4%) participants were randomly allocated to either the 50% resveratrol group (*n* = 28) or the 50% placebo group (*n* = 28). During the study, four participants from both the placebo and resveratrol groups withdrew from the trial. In the resveratrol group, two patients withdrew due to pregnancy, and two discontinued the intervention. In the placebo group, one patient withdrew for personal reasons, two patients withdrew due to pregnancy, and one patient discontinued the intervention. Consequently, the study included 24 (50%) participants in each group, as demonstrated by the distribution of participants throughout the trial in the Consolidated Standards of Reporting Trials (CONSORT) diagram depicted in Fig. 1.

### Baseline clinical and endocrine characteristics of the study population

No significant differences were observed between the two groups regarding mean age, BMI, duration of infertility, menstruation, menstrual cycle duration, or hormonal profiles on the day of study initiation (*P* > 0.05 for all; Table 2).

**Table 2:** Baseline characteristics and endocrine profiles of the participants

Variables	Placebo group (*n* = 24) Mean ± SD	Resveratrol group (*n* = 24) Mean ± SD	*P* value
Age (years)	30.75 ± 5.169	29.38 ± 5.515	0.3775
BMI (kg/m^2^)	26.48 ± 4.010	26.38 ± 3.042	0.9165
Duration of infertility (years)	5.292 ± 2.774	5.042 ± 2.349	0.7377
Duration of menstruation (day)	6.458 ± 1.641	6.583 ± 1.283	0.7701
Duration of menstrual cycle (day)	39.96 ± 7.369	41.46 ± 6.903	0.4704
FSH (µIU/mL-baseline)	4.135 ± 1.286	4.670 ± 1.686	0.2220
LH (µIU/mL-baseline)	6.818 ± 4.223	7.111 ± 3.980	0.9677
Prolactin (mIU/l-baseline)	268.6 ± 127.3	277.3 ± 125.2	0.8122
TSH (µIU/mL-baseline	2.42 ± 1.045	2.681 ± 1.279	0.4423
Testosterone (ng/ml- baseline)	0.7054 ± 0.3864	0.7446 ± 0.345	0.9298
AMH (ng/ml- baseline)	7.908 ± 3.381	7.348 ± 3.270	0.5622

The data are presented as the means ± SDs. Unpaired t tests were used to analyze the differences among groups. A *P* value < 0.05 was considered to indicate statistical significance

BMI, Body mass index; FSH, Follicle-stimulating hormone; LH, Luteinizing hormone; PRL, Prolactin; TSH, Thyroid-stimulating hormone; AMH, Anti-Müllerian hormone

### Primary outcome: effects of resveratrol on OS markers

The findings revealed that the TOS and OSI were significantly lower in the treatment group than in the placebo group (*P* < 0.05; Table 3). Additionally, the TAC in FF, as assessed by the FRAP assay, significantly increased between the two groups (*P* < 0.05; Table 3).

**Table 3:** Comparison of OS markers in follicular fluid between the treatment and placebo groups

FF oxidative statues	Placebo group (*n* = 24)	Resveratrol group (*n* = 24)	*P* value
FF TAC (mmol Fe2 +/l)^a^	1.127 ± 0.3789	1.487 ± 0.3257	0.0009***
FF TOS (µmol H2O2Eq/l)^b^	2.330(1.333–3.370)	1.885(0.2490–2.422)	0.0142*
FF OSI (µmol Eq H2O2/mmol Fe2+)^a^	2.51 ± 1.908	1.074 ± 0.9181	0.0039**

FF, follicular fluid; TAC, total antioxidant capacity; TOS, total oxidant status; OSI, oxidative stress index. A *P* value < 0.05 was considered to indicate statistical significance. ***P < 0.001, **P < 0.01, *P < 0.05

^a^ Unpaired t tests were used to analyze the differences among groups. The data are presented as the means ± SDs.

^b^ Mann–Whitney test was used to analyze the differences among groups. The data are presented as the median (25th–75th percentile)

### Secondary outcomes

#### Effects of resveratrol on mRNA expression levels

**Fig. 2:**
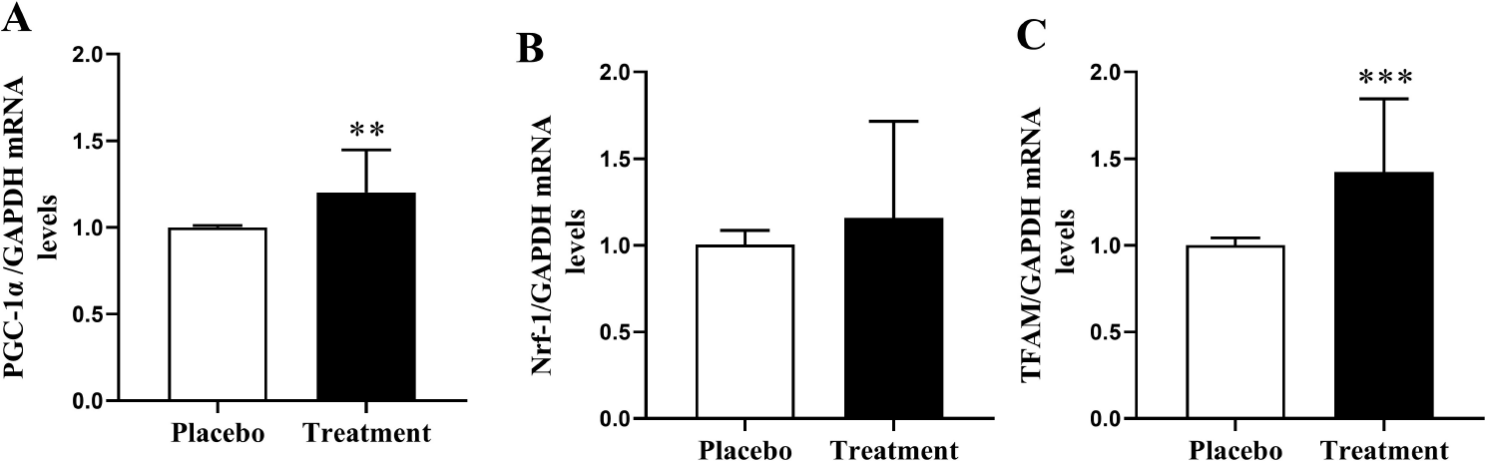
The expression of PGC-1α (**A**), Nrf-1 (**B**), and TFAM (**C**) in GCs from the resveratrol and placebo groups. (**A**, **C**) The results revealed that the expression of PGC-1α and TFAM was significantly greater in the resveratrol group (*P* < 0.05). (**B**) The increase in Nrf-1 levels did not reach statistical significance between the two groups (*P* > 0.05). The treatment group received a daily dose of 800 mg/day of resveratrol. The data are presented as the means ± SDs. ***P < 0.001, **P < 0.01. (Mann‒Whitney test). PGC-1α, peroxisome proliferator-activated receptor gamma coactivator; Nrf-1, nuclear respiratory factor 1; TFAM, mitochondrial transcription factor A

 We hypothesized that resveratrol might also exert regulatory effects on downstream factors within the PGC-1α pathway. qRT‒PCR measurements indicated that the expression levels of mitochondrial biogenesis-related genes, such as PGC-1α and TFAM, were significantly elevated in the treatment group compared to those in the placebo group (*P* = 0.0032 and *P* = 0.0003; Fig. 2A C, respectively). Although there was an increase in Nrf-1 mRNA expression in the treatment group, this increase did not reach statistical significance (*P* = 0.0611; Fig. 2B). These results provide strong evidence that resveratrol enhances the expression of downstream factors of PGC-1α at the mRNA level by activating the PGC-1α signaling pathway.

#### Effects of resveratrol on the protein expression levels

**Fig. 3:**
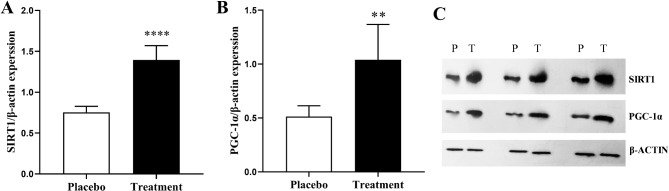
The protein expression levels of SIRT1 and PGC-1α in GCs from the placebo and resveratrol groups. (**A**, **B**) Western blot analysis was performed to assess the protein expression of SIRT1 and PGC-1α, which was subsequently normalized to that of β-actin. The protein expression of SIRT1 and PGC-1α was significantly greater in the treatment group than in the control group (*P* < 0.05). (**C**) Blots of SIRT1, PGC-1α, and β-actin. The treatment group received a daily dose of 800 mg/day of resveratrol. The data are presented as the means ± SDs. ****P < 0.0001, **P < 0.01 (Unpaired t test). SIRT1, Sirtuin 1; PGC-1α, Peroxisome proliferator-activated receptor gamma coactivator

Resveratrol upregulated the protein expression of SIRT1 and PGC-1α in GCs (Fig. 3). The protein levels of SIRT1 and PGC-1α were significantly greater in the treatment group than in the placebo group (*P* < 0.0001; *P* = 0.0036; Fig. 3A and B, respectively).

#### Effects of resveratrol on the mtDNA copy number and intracellular ATP content

**Fig. 4:**
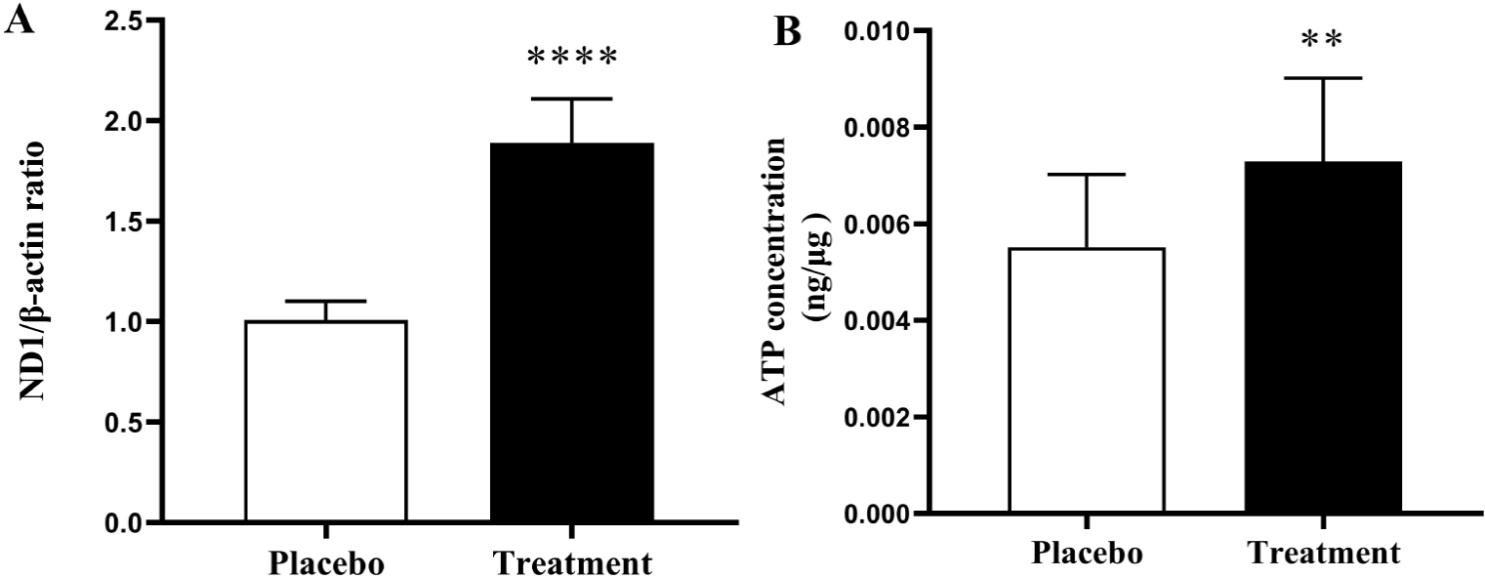
Comparison of mtDNA copy number and intracellular ATP content between the two groups. (**A**) The relative mtDNA copy numbers were significantly greater in the treatment group than in the control group (*P* < 0.05). (**B**) A comparison of the intracellular ATP content in GCs between the resveratrol and placebo groups revealed a significant increase in the resveratrol group (*P* < 0.05). The treatment group received a daily dose of 800 mg/day of resveratrol. The data are presented as the means ± SDs. ****P < 0.0001, **P < 0.01 (Unpaired t test). ND1, NADH-dehydrogenase subunit; ATP, adenosine triphosphate

In contrast to the placebo group, the treatment group exhibited an elevated mtDNA copy number (*P* < 0.0001; Fig. 4A). To determine the effect of resveratrol treatment on mitochondrial biogenesis and function, we measured the intracellular ATP concentration, which was found to increase in the intervention group (*P* = 0.0014; Fig. 4B). These findings collectively suggest that resveratrol enhances mitochondrial biogenesis in the GCs of patients with PCOS.

#### Evaluation of ovarian stimulation parameters and ART results

According to the analysis of ovarian stimulation parameters and ART outcomes, resveratrol therapy significantly improved the oocyte maturity rate and percentage of high-quality embryos (*P* < 0.05). However, there were no significant differences between the two groups in terms of the number of retrieved oocytes, the number of MII oocytes, the total number of embryos, the number of fertilized oocytes, or the fertilization rate (*P* > 0.05) (Table 4).

**Table 4:** Comparison of clinical outcomes between the treatment and placebo groups

Variables	Placebo group (*n* = 24)	Resveratrol group (*n* = 24)	*P* value
Number of retrieved oocytes ^b^	24.50(14.25–32.75)	24.50(16.50–32.00)	0.9999
Number of MII (mature) oocyte ^a^	16.5 ± 8.367	19.17 ± 7.149	0.2413
Rate of maturity oocyte ^a^	64.73 ± 15.65	78.04 ± 10.56	0.0012**
Fertilized oocyte ^a^	11.25 ± 6.435	14 ± 5.572	0.1203
Rate of fertilization ^b^	68.99(61.78–79.44)	70.90(66.68–86.28)	0.2839
Number of embryos ^a^	13.38 ± 6.579	16.21 ± 6.22	0.1321
Rate of high-quality embryo ^a^	63.54 ± 15.79	75.23 ± 5.648	0.0013**

^a^ Unpaired t tests were used to analyze the differences among groups. A *P* value < 0.05 was considered to indicate statistical significance. The data are presented as the means ± SDs. **P < 0.01

^b^ Mann–Whitney test was used to analyze the differences among groups. A *P* value < 0.05 was considered to indicate statistical significance. The data are presented as the median (25th–75th percentile)

**Fig. 5:**
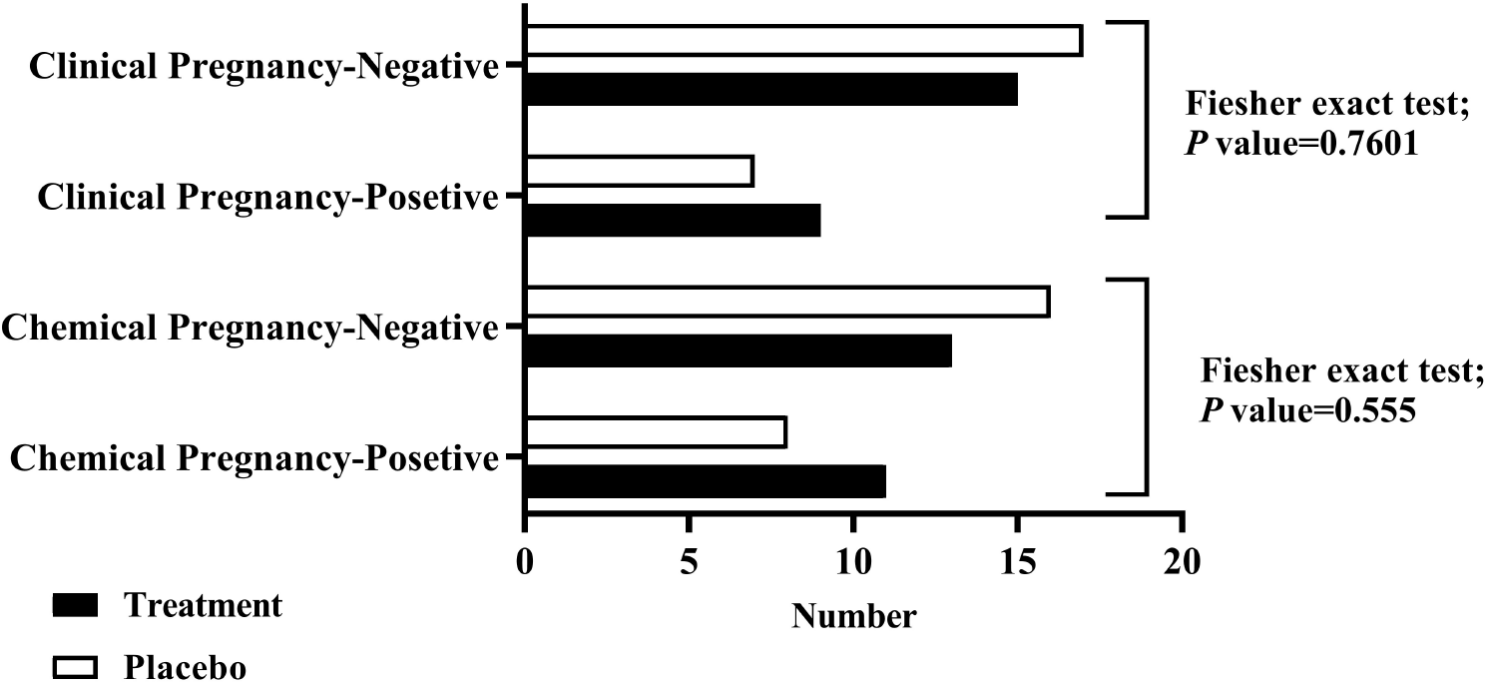
Comparison of the chemical and clinical pregnancy rates between the resveratrol and placebo groups. In the resveratrol group, the chemical pregnancy rate was 45.83% (11/24), while it was 33.33% (8/24) in the placebo group (Fisher’s test; two-tailed *P* = 0.5556). Furthermore, the clinical pregnancy rate was 37.50% (9/24) in the resveratrol group and 29.17% (7/24) in the placebo group (Fisher’s test; two-tailed *P* = 0.7601). Placebo: *n* = 24, Treatment: *n* = 24

Furthermore, there were no significant differences detected between the resveratrol and placebo groups in terms of chemical pregnancy or clinical pregnancy rates (45.83% (11/24) vs. 33.33% (8/24); two-tailed *P* = 0.5556; 37.50% (9/24) vs. 29.17% (7/24); two-tailed *P* = 0.7601; Fig. 5).

## Discussion

In the context of PCOS, women face hurdles in the progression of oocyte development, resulting in decreased rates of fertilization and challenges in the implantation of embryos [36]. Recent research has highlighted the correlation between increased OS and the progression of PCOS and mitochondrial dysfunction [37]. GCs contribute to the increased accumulation of FF surrounding the oocyte within the developing follicle. Imbalances between antioxidant elements and ROS in FF can potentially have detrimental effects on oocyte quality, fertilization, and embryo development by disrupting the balance within the follicular microenvironment, potentially leading to infertility in patients with PCOS [38]. Considering the OS status of these women, antioxidant therapy may offer potential benefits for individuals with PCOS [6]. In several animal studies, resveratrol treatment was shown to ameliorate oxidative damage by reducing TOS and increasing TAC [14, 39–41]. In the present study, a comparison of the OS in FF revealed that the TOS and OSI were significantly lower in the resveratrol group than in the placebo group. TAC serves as an indicator of the abundance of antioxidants actively involved in neutralizing free oxygen radicals in the body. In this study, the levels of TAC were significantly greater in the resveratrol group than in the placebo group, corroborating findings from another clinical trial [25]. The evidence mentioned above indicates potential therapeutic applications of resveratrol within a clinical context. We also focused on exploring the impact of resveratrol treatment on mitochondrial biogenesis in GC patients with PCOS, particularly through activating the SIRT1-PGC-1α signaling pathway. The results of our study can be summarized as follows: (i) The protein levels of SIRT1 and PGC1-α were significantly elevated in response to resveratrol treatment compared to those in the placebo group; (ii) The administration of resveratrol led to an increase in the expression of pivotal genes essential for mitochondrial biogenesis, specifically PGC1-α, Nrf-1, and TFAM mRNA, indicating a positive pharmacological effect;  (iii) Interestingly, resveratrol treatment was associated with an increase in mtDNA copy numbers and intracellular ATP content; and (iv) resveratrol ameliorated ART outcomes. Several studies have explored the potential of resveratrol to improve mitochondrial biogenesis in the context of reproduction [42]. The influence of resveratrol on mitochondrial biogenesis in human GCs could explain the positive impacts of this polyphenol on female reproductive system physiology [20]. In bovine models, resveratrol has been shown to stimulate mitochondrial biogenesis and enhance energy metabolism in aged oocytes. Additionally, this treatment has been shown to facilitate bovine oocyte maturation and promote embryonic development [43]. Individuals with PCOS exhibit irregular mitochondrial structure, function, and gene expression within their follicles. These anomalies impact follicular growth, ovulation, and fertilization [37]. Mitochondrial biogenesis in women with PCOS has been explored in limited studies. A study revealed significant mitochondrial suppression in the muscles of women with PCOS. The study indicated a reduction in the expression of the PGC-1α gene, which was linked to decreased expression of OXPHOS genes [44]. In the PCOS cohort, there was a decrease in the gene expression of PGC-1α, accompanied by a high level of methylation in the PGC-1α promoter region. Consequently, methylation of the PGC-1α promoter suppresses the transcription of PGC-1α, leading to a decrease in the rate of mitochondrial biogenesis [45]. A previous study reported that resveratrol can activate PGC-1α and enhance the expression of factors involved in mitochondrial biogenesis [10]. In this study, we noted that the administration of resveratrol contributed to the enhancement of mitochondrial biogenesis by elevating the protein levels of SIRT1 and PGC-1α, as did the upregulation of downstream factors, including Nrf-1 and TFAM, which are associated with increased mitochondrial biogenesis in patients with PCOS. The quantity of mtDNA remains constant, and the preservation of appropriate mtDNA copy numbers is important for sustaining mitochondrial function and cellular growth [46]. Previous research has connected changes in mtDNA copy number with reproductive disorders, including endometrial cancer, premature ovarian aging, and PCOS [47–49]. Recent findings indicate that quantitative alterations in mtDNA are linked to the occurrence and progression of PCOS [49]. Most existing investigations have quantified mtDNA levels in the blood of patients with PCOS, and limited research has been carried out in muscle [49–51]. One study reported decreased mtDNA copy numbers in women with PCOS relative to those without PCOS [49]. Conversely, another study reported no significant associations between mtDNA copy number and PCOS [50]. A prior animal investigation demonstrated that resveratrol led to an increase in mtDNA copy numbers within oocytes [42]. There is also growing evidence that optimal mtDNA copy numbers and sufficient ATP levels are essential for ensuring proper follicular maturation and blastocyst development [52, 53]. Furthermore, we measured the mtDNA copy number and ATP levels after resveratrol treatment, which were notably elevated in the resveratrol group. In line with our results, resveratrol increased ATP levels in oocytes from aged cows and pigs [42, 54], suggesting its potential for reversing mitochondrial dysfunction and enhancing mitochondrial biogenesis. A recent in vitro study investigated the effects of supplementation with various antioxidants and revealed that it enhanced the developmental competence of oocytes and improved both the quantity and quality of embryos [55]. According to several animal model studies, resveratrol activates SIRT1, thereby improving the biosynthesis of oocyte mitochondria and enhancing the developmental potential of oocytes [56]. More importantly, in vivo, resveratrol administration has been found to influence the maturation of bovine oocytes and embryonic development post in vitro fertilization (IVF) by stimulating progesterone secretion and offering antioxidant properties [43]. Resveratrol supplementation inhibits follicular atresia and increases the reserve of ovarian follicles and the lifespan of ovaries. This supplementation also improves the quantity and quality of oocytes [57, 58]. In addition, in vivo studies have shown the antioxidative properties of resveratrol and its capacity to normalize ovarian morphology [59]. A recent laboratory experiment indicated that the use of resveratrol as a supplement in in vitro maturation (IVM) medium improved oocyte maturation and quality, fertilization rates, and blastocyst formation in both aged mice and humans by improving spindle morphology, chromosome alignment, and mitochondrial function [60]. Several clinical trials have explored the impact of resveratrol on ART outcomes. A previous study reported that resveratrol significantly improved the percentage of high-quality oocytes and embryos [18]. Consistent with our study, resveratrol treatment significantly enhanced the rate of oocyte maturation and the percentage of high-quality embryos. Women with PCOS typically produce a greater number of oocytes during stimulation in an IVF cycle, but the quality of these oocytes is often poor [61]. Previous studies have indicated that the poorer ART outcomes in patients with PCOS than in those without PCOS are primarily due to the lower quality and maturity of oocytes in these patients [6]. This evidence suggests that impaired oocyte maturation and poor oocyte quality in patients with PCOS may result in compromised embryo development [38]. In line with our findings, multiple prospective randomized studies in infertile women undergoing IVF-ET cycles have shown that oral antioxidant therapy increased oocyte maturation, enhanced oocyte quality, and resulted in a greater ratio of high-quality embryos [62–64]. Research has also confirmed that the quality of embryos positively affects implantation and pregnancy outcomes [65]. Along with more oocyte maturation and higher embryo quality, we found that resveratrol enhanced fertilization, chemical, and clinical pregnancy rates; however, the difference between the groups was not statistically significant. No significant differences were observed in the clinical data, most likely due to the small sample size. In addition, fertilization and pregnancy outcomes can be significantly affected by the quality and maturity of gametes, uterine receptivity, and competence of the embryologist [66]. In this context, our findings align with those of previous clinical trials [18, 19, 64, 67]. However, some researchers reported contradictory results, highlighting the detrimental effect of resveratrol on the clinical pregnancy rate [68]. In their study, resveratrol was administered during the luteal phase of the treatment cycle, and this adverse effect was likely attributed to its impact on the suppression of decidual senescence. The discrepancy between our trial and another study, which used a low dose of resveratrol (200 mg daily), may be due to the various treatment durations, heterogeneous populations, retrospective design, and different IVF protocols used [67]. More specifically, in our trial, resveratrol was administered before and during ovarian stimulation, and intake was discontinued on the day of oocyte retrieval. The 60-day pretreatment duration was determined by considering the average duration of oogenesis and the stages of follicle development, during which oocytes undergo initial maturation in the ovary [69]. The resveratrol dose used in our study has been demonstrated to be safe and well tolerated in previous research [70, 71]. Additionally, studies have revealed that consuming resveratrol at doses ranging from 700 to 1000 mg/kg body weight/day is not toxic [72–74]. Several scientific studies have also reported that a daily dose of up to 1000 mg of resveratrol has antioxidant effects on patients with PCOS and other medical conditions [73, 75–79]. In our study, the effective dose and duration of resveratrol supplementation were derived from a previous study [25]. Oral supplementation with resveratrol has been shown to reduce OS markers (one of the primary goals of this study). Additionally, two clinical trials [18, 19] have investigated the effects of resveratrol at a dosage of 800 mg/day on ART outcomes in patients with PCOS, similar to our study population. However, no studies have examined the impact of resveratrol on mitochondrial biogenesis factors; furthermore, this dose and duration were chosen for this study. The strengths of our study lie in its design, prospective nature, and novel investigation of the effect of resveratrol on mitochondrial biogenesis in the GCs of women with PCOS undergoing assisted reproduction. Additionally, the design of our triple-blind randomized clinical trial with parallel groups (treatment and placebo), along with the inclusion of patients with the same baseline parameters in both groups, remarkably enhances the findings of this study. This study has several limitations and weaknesses. First, the follow-up period was relatively short, and the sample size was small. Additionally, time constraints prevented the evaluation of late pregnancy outcomes, such as live birth. Therefore, future studies with longer follow-ups and larger sample sizes are necessary to analyze pregnancy outcomes comprehensively. Further research with various doses and durations is needed to confirm our findings. In addition, due to limited resources, effectively managing and ensuring that participants adhered to a control diet for 60 days was challenging in this study. It is suggested that future studies utilize both the three-day food recall questionnaire and the Nutritionist IV program (modified for Iranian food composition) to calculate food intake and dietary intake, respectively. In conclusion, our findings indicate that 60 days of supplementation with 800 mg/day resveratrol enhances the expression of specific genes and proteins linked to mitochondrial biogenesis, improving mtDNA copy number and increasing ATP content in GCs. Moreover, our findings suggest that resveratrol may also modulate OS marker levels in the FF of patients, thus protecting against OS damage. Ultimately, resveratrol intervention influences oocyte maturity and embryo quality. These findings reinforce the hypothesis that resveratrol potentially improves mitochondrial biogenesis in GCs and may enhance ART outcomes in patients with PCOS.

## Data Availability

All data supporting the findings of this study are available within the paper.

## Abbreviations

AMH: Anti-Müllerian Hormone
ART: Assisted Reproductive Technology
BMI: Body Mass Index
cDNA: Complementary DNA
CONSORT: Consolidated Standards of Reporting Trials
ECL: Enhanced Chemiluminescence
FF: Follicular Fluid
FSH: Follicle-Stimulating Hormone
GAPDH: Glyceraldehyde-3‐Phosphate Dehydrogenase
GCs: Granulosa Cells
hCG: Human Chorionic Gonadotropin
ICSI: Intracytoplasmic Sperm Injection
IRCT: Iranian Registry of Clinical Trials
IVM: In Vitro Maturation
IVF: In Vitro Fertilization
LH: Luteinizing Hormone
mtDNA: Mitochondrial DNA
ND1: NADH-Dehydrogenase Subunit 1
Nrf-1: Nuclear Respiratory Factor 1
OHSS: Ovarian Hyperstimulation Syndrome
OS/OSI: Oxidative Stress/Oxidative Stress Index
PBS: Phosphate-Buffered Saline
PCOM: Polycystic Ovary Morphology
PCOS: Polycystic Ovary Syndrome
PGC-1α: Peroxisome Proliferator-Activated Receptor Gamma Coactivator
PRL: Prolactin
PVDF: Polyvinylidene Difluoride
qPCR: Quantitative Polymerase Chain Reaction
qRT–PCR: Quantitative Real-Time Polymerase Chain Reaction
SD: Standard deviation
SIRT1: Sirtuin 1
TFAM: Mitochondrial Transcription Factor A
TAC: Total Antioxidant Capacity
TOS: Total Oxidant Status
TSH: Thyroid Stimulating Hormone
